# Descriptive study of 100 patients with a diagnosis of psychosis treated with Paliperidone Palmitate 6-Month Long-Acting Injectable.

**DOI:** 10.1192/j.eurpsy.2024.796

**Published:** 2024-08-27

**Authors:** J. Mesones, A. Miller, N. Bautista, J. Dieguez, I. Calero, A. Griñant

**Affiliations:** ^1^HOSPITAL UNIVERSITARIO TORREVIEJA, ALICANTE, Spain

## Abstract

**Introduction:**

Psychotic disorders are serious mental illnesses that require long-term antipsychotic treatment that provides sufficient efficacy, safety and therapeutic adherence. The latter is an essential factor that must be emphasized in clinical practice in order to avoid relapses. On this occasion, we have the need to know the long-term impact on our clinical practice and on the evolution of patients after the change in formulation of paliperidone palmitate 1 (PP1M) and 3 Month long-acting injectable antipsychotic (PP3M) to paliperidone palmitate 6 Month (PP6M).

**Objectives:**

The present study describes a sample of patients with severe mental disorders (n= 100) treated with six-monthly paliperidone palmitate (PP6M) studying the diagnoses, socio-demographic characteristics, number of relapses, tolerability and treatment adherence of patients.

**Methods:**

Prospective descriptive study with a sample selected by non-probabilistic consecutive sampling, retrospective type, in a time interval of 15 month (n= 100 outpatients). The patients selected were all those who received 6 monthly paliperidone palmitate treatment from May 2022 to September 2023. A descriptive analysis was performed. Mean and standard deviation were calculated for quantitative variables and N and percentage for categorical variables.

**Results:**

Prospective study with consecutive sampling of 100 outpatients (62% men, 38% women; mean age 48 years) diagnosed with psychosis (76 % Schizophrenia, 21 % Unspecified psychosis, 3 % Delusional disorder) those who are administered PP6M long-acting injectable antipsychotic previously treated with PP1M (35%) and PP3M (65%).

After 15 months of the study, 4 patients (4%) have suffered a relapse, one of them (1%) requiring hospitalization. 5 patients (4%) declined to continue PP6M and have returned to their previous injectable. 1 patient (1%) has died of unknown causes outside the treatment. 90 patients continue treatment with PP6M (90% retention rate). 54 patients maintain antipsychotic monotherapy (54%). No additional adverse effects were reported after switching to PP6M. The subjective perception of satisfaction after the switch to PP6M by patients and caregivers was very high.

**Conclusions:**

The present real clinical practice study shows that PP6M could be an effective and well tolerated treatment in patients with severe mental disorder, for patients diagnosed with psychosis, with a high rate of relapse prevention and high rates of compliance. Changing treatment from PP1M or PP3M to PP6M could help patients with severe mental disorder to normalize their lives and functionality.

**Disclosure of Interest:**

None Declared

